# Effects of n-3 PUFAs on breast cancer cells through their incorporation in plasma membrane

**DOI:** 10.1186/1476-511X-10-73

**Published:** 2011-05-12

**Authors:** Paola A Corsetto, Gigliola Montorfano, Stefania Zava, Ilaria E Jovenitti, Andrea Cremona, Bruno Berra, Angela M Rizzo

**Affiliations:** 1 Dipartimento di Scienze Molecolari Applicate ai Biosistemi, Università degli Studi di Milano, Italy

## Abstract

**Background:**

PUFAs are important molecules for membrane order and function; they can modify inflammation-inducible cytokines production, eicosanoid production, plasma triacylglycerol synthesis and gene expression. Recent studies suggest that n-3 PUFAs can be cancer chemopreventive, chemosuppressive and auxiliary agents for cancer therapy. N-3 PUFAs could alter cancer growth influencing cell replication, cell cycle, and cell death. The question that remains to be answered is how n-3 PUFAs can affect so many physiological processes. We hypothesize that n-3 PUFAs alter membrane stability, modifying cellular signalling in breast cancer cells.

**Methods:**

Two lines of human breast cancer cells characterized by different expression of ER and EGFR receptors were treated with AA, EPA or DHA. We have used the MTT viability test and expression of apoptotic markers to evaluate the effect of PUFAs on cancer growth. Phospholipids were analysed by HPLC/GC, to assess n-3 incorporation into the cell membrane.

**Results:**

We have observed that EPA and DHA induce cell apoptosis, a reduction of cell viability and the expression of Bcl2 and procaspase-8. Moreover, DHA slightly reduces the concentration of EGFR but EPA has no effect. Both EPA and DHA reduce the activation of EGFR.

N-3 fatty acids are partially metabolized in both cell lines; AA is integrated without being further metabolized. We have analysed the fatty acid pattern in membrane phospholipids where they are incorporated with different degrees of specificity. N-3 PUFAs influence the n-6 content and vice versa.

**Conclusions:**

Our results indicate that n-3 PUFA feeding might induce modifications of breast cancer membrane structure that increases the degree of fatty acid unsaturation. This paper underlines the importance of nutritional factors on health maintenance and on disease prevention.

## Background

Breast cancer is the most common cancer among women worldwide, with an estimated 1.4 million new breast cancer cases only in 2008. Epidemiologic and experimental studies suggest that dietary fatty acids influence the development and subsequent progression of breast cancer [1-3]. The role that long-chain n-3 polyunsaturated fatty acids (PUFAs), eicosapentaenoic acid (EPA, 20:5n-3) and docosahexaenoic acid (DHA, 22:6n-3), play in the aetiology of cancer has been highlighted by animal experiments and in vitro studies [4,5]. A number of mechanisms have been proposed for the anticancer actions of n-3 PUFAs. The most prominent mechanism for the chemopreventive action of n-3 PUFAs is their suppressive effect on the production of arachidonic acid (AA)-derived prostanoids, particularly prostaglandin E_2 _(PGE_2_), which has been implicated in the immune response to inflammation, cell proliferation, differentiation, apoptosis, angiogenesis and metastasis [6].

The n-3 PUFAs might alter the growth of tumour cells by influencing cell replication, by interfering with components of the cell cycle or by increasing cell death either by way of necrosis or apoptosis [7,8]. For example, these fatty acids are involved in regulating the tumour p53 proapoptotic signal and superoxide dismutase (SOD) levels, telomere shorting and tumour angiogenesis [9]. In vitro treatment with DHA arrested cell-cycle progression in human-derived breast cancer and malignant melanoma cells [10,11]. Similarly, in vitro treatment with EPA is reported to arrest the growth of K-562 human leukemic and many other cancer cells accompanied by down-regulation of cyclin expression in some instances [12-14].

In addition, recent studies of human breast cancer have shown that n-3 PUFAs up-regulate syndecan 1 (SDC-1), which has been shown to play a role in cell adhesion [15,16], inhibit matrix metalloproteinases [17] and decrease invasion of tumour cells. SDC-1 induces apoptosis in myeloma cells and some studies suggest a similar property in breast cancer cells [18,19]. The transcriptional pathway for the n-3 PUFA regulation of SDC-1 expression involves the nuclear hormone receptor peroxisome proliferator-activated receptor gamma (PPARγ) [20]. Moreover n-3 PUFAs down-regulate the expression of HER2/*neu*, a well characterized oncogene that plays a key role in aetiology, progression and chemosensitivity of various types of human cancer in which this oncogene is over-expressed. HER2/*neu *encodes transmembrane tyrosine kinase orphan receptor p185^Her2/neu^, which regulates biological functions including cellular proliferation, differentiation, motility and apoptosis [21].

Nevertheless the mechanism by which n-3 PUFAs inhibit the growth of breast cancer cells is not well understood, but it has been suggested that these fatty acids might change the fluidity and structure of the cell membrane. In fact, changes in the structural characteristics of the plasma membrane in mammalian cells can modify the activity of proteins that function as ion channels, transporters, receptors, signal transducers or enzymes [21-25].

In this study, we have investigated the impact of EPA, DHA and AA on breast cancer cell growth, on cell signalling in apoptosis and on epidermal growth factor receptor (EGFR) activity. We hypothesize that the alteration of cellular cycle, of gene expression and the induction of apoptosis determined from n-3 PUFAs are also a consequence of membrane architecture modifications. For these reasons we have analyzed PUFA incorporation in breast cancer membrane and their PL-specific enrichment.

## Methods

### Cell lines and culture conditions

Human breast cancer cell lines MDA-MB-231 (ER-negative) and MCF-7 (ER-positive) were kindly provided by Dr P. Degan from the IST (Italian National Cancer Research Institute, Genoa Italy, Laboratory of Molecular Mutagenesis and DNA Repair). Both cell lines are derived from human mammary adenocarcinoma; the MCF7 line retains several characteristics of differentiated mammary epithelium, including the ability to process estradiol via cytoplasmic estrogen receptors. The MDA-MB-231 cells over-express EGFR.

These cell lines were maintained in DMEM (Gibco-BRL, Life Tecnologies Italia srl, Italy) supplemented with 10% fetal bovine serum (FBS), 100 U/ml penicillin, 100 mg/ml streptomycin and 2 mM glutamine.

Medium for treatments (MFT) was DMEM supplemented with 10% FBS. Cells were grown at 37°C in a 5% CO_2 _atmosphere with 98% relative humidity.

### PUFAs

EPA (*cis*-5,8,11,14,17-eicosapentaenoic acid sodium salt), DHA (*cis*-4,7,10,13,16,19-docosahexaenoic acid sodium salt) and AA (arachidonic acid sodium salt) were purchased from Sigma-Aldrich, USA. The PUFAs were dissolved in ethanol and stored at -80°C under nitrogen gas.

### Antibodies

The mouse monoclonal anti-Bcl2 antibody (Santa Cruz Biotechnology Inc., Santa Cruz, CA, USA) and the C20 goat polyclonal anti-procaspase-8 p18 antibody were used to study the n-3 PUFA induction of the apoptosis process. The 1005 rabbit polyclonal anti-EGFR antibody and the 11C2 mouse monoclonal anti-pEGFR antibody (Santa Cruz Biotechnology Inc., Santa Cruz, CA, USA) were used to investigate the alterations of EGFR receptors after treatment with PUFAs. The monoclonal anti-actin (AC-40) antibody (Sigma-Aldrich, USA) was used to normalize gel loading.

Bound primary antibodies were visualized by secondary horseradish peroxidase (HRP)-linked antibodies (Santa Cruz Biotechnology Inc., Santa Cruz, CA, USA) and immunoreactivity was assessed by chemiluminescence (ECL, Amersham).

### Cell viability assay

The numbers of viable cells exposed to fatty acids were evaluated by the MTT (3-(4,5-dimethylthiazol-2-yl)-2,5-diphenyltetrazolium bromide) colorimetric assay [26]. Initially, cells were seeded and cultured in 96-well plates for 48 h to allow adhesion to the plate and to reach 50-60% confluence. After this period, the culture medium was changed to the experimental medium supplemented with EPA or DHA or AA then cultured for 72 h. We studied the effects of different concentrations of PUFAs (50-300 μM). The final concentration of ethanol (<1%) in the culture medium had no antiproliferative effect on any cell line tested; therefore, 10 μl of MTT stock solution (5 mg/ml in PBS, pH 7.5) was added to each well and incubated for 4 h as a control. Then 100 μl of solubilizing solution (10% SDS in 0.01M HCl) was added and incubated overnight. Plates were read at 540 nm in a plate reader. All reagents were purchased from Sigma-Aldrich, USA. Data points represent the mean of eight wells and the results are expressed as relative growth rate (RGR) in comparison to controls that were exposed to a concentration of ethanol equal to that in the samples exposed to fatty acids.

### Cell treatment

Cell culture experiments were done with the MDA-MB-231 and MCF-7 cell lines to determine the concentrations of EPA (230 μM), DHA (200 μM), required to inhibit growth by 20-30%, and AA (200 μM). Cells were seeded at 1.5 × 10^4 ^cells/cm^2 ^for MDA-MB-231 and at 3 × 10^4 ^cells/cm^2 ^for MCF-7 in 18 ml of medium containing 10% FBS and allowed to adhere for 48 h, then the medium was replaced with 18 ml of fresh medium (DMEM, 10% FBS) containing the experimental fatty acids and incubated for 72 h without changing the medium. Experiments included untreated cells that were not exposed to any exogenous fatty acids but to an equal content of ethanol during the incubation to serve as controls. After 72 h, cells were harvested using trypsin-EDTA and centrifuged at 900 rpm for 10 min. The supernatant was removed and the pellets were subjected to lipid analysis.

For western blot analysis, cells were harvested by scraping in phosphate-buffered saline containing 0.4 mM Na_3_VO_4_. Cells were centrifuged and then suspended in 1.4 ml of lysis buffer (1% Triton X-100, 10 mM Tris buffer, pH 7.5, 150 mM NaCl, 5 mM EDTA, 1 mM Na_3_VO_4_, 1 mM phenylmethylsulfonyl fluoride, 75 mU/ml aprotinin), kept on ice for 20 min and then disrupted by 10 strokes in a tight-fitting Dounce homogenizer. The cell lysate was centrifuged (5 min at 1300 *g*) and the supernatant was transferred to an eppendorf tube. Total protein was determined by the Lowry assay [27].

### Lipid composition analysis

Cell lipids were extracted with three different chloroform/methanol mixtures (1:1, 1:2 and 2:1, v/v) and partitioned with a theoretical upper phase (chloroform/methanol/water, 47:48:1, by vol.) and then with water. The organic phase was dried and then suspended in chloroform/methanol (2:1, v/v) for the analysis of total and PL fatty acids.

Purification of single PL moieties was achieved with an HPLC-ELSD system (Jasco, Japan) equipped with one pump, a SCL-10 Advp, a degasser module and a Rheodyne manual injector with 20 μl sample loop and a column (length 250 mm, I.D 4.6 mm and film thickness 5 μm) packed with silica normal-phase LiChrospher Si 60 (LiChroCART 250-4, Merck, Darmstadt, Germany).

The chromatographic separation was achieved with a linear binary gradient of 0% B to 100% B in 14 min and then 100% B for 9 min. The total chromatographic run time was 40 min/sample; 23 min analysis, 12 min to restore initial conditions and 5 min for re-equilibration. Eluent A was chloroform/methanol/water (80:19.5:0.5, by vol.) and eluent B was chloroform/methanol/water (60:34:6, by vol.) and the flow rate of the eluent was 1.0 ml/min. An evaporative light-scattering detector (ELSD) was used to detect and quantify the separated PL species.

After elution, the eluate was split with one part going to the detector and nine parts to a Gilson fraction collector model 201 to collect the different PL classes for GC analysis.

Total fatty acids and PL fatty acids were determined as methylesters by gas chromatography (GC). The methyl esters were obtained by reaction with 3.33% (w/v) sodium methoxide in methanol and injected into an Agilent Technologies (6850 series II) gas chromatograph equipped with a flame ionization detector (FID) and a capillary column (AT Silar) (length 30 m, film thickness 0.25 μm). The carrier gas was helium, the injector temperature was 250°C, the detector temperature was 275°C, the oven temperature was set at 50°C for 20 min and then increased to 200°C at 10°C min^-1 ^for 20 min.

### Western blot analysis for Bcl2, caspase-8, EGFR and pEGFR

Control and treated cell lysates (100 μg protein/lane) were separated by SDS-PAGE (10% polyacrylamide gel), transferred to a polyvinylidene difluoride (PVDF) membrane and analysed by western blot with anti-Bcl2 (1:100), anti-caspase-8 (1:500) and β-actin (1:1800) antibodies. The PVDF membrane was blocked for 1 h in blocking buffer 5% (w/v) dried non-fat milk in Tris-buffered saline (T-TBS: 10 mM Tris-HCl, pH 7.5, 150 mM NaCl, 0.1% (v/v) Tween^®^20) followed by incubation with an appropriate primary antibody in blocking buffer at room temperature for 2 h. The blots were washed with T-TBS and then incubated with the proper secondary antibody in blocking buffer at room temperature for 1 h. The protein bands were visualized using ECL western blot detection reagents (PerkinElmer, USA).

For the analysis of EGFR and p-EGFR, cells treated or not with DHA and EPA were cultured in MFTs supplemented with 10 nM EGF (Sigma-Aldrich, St. Louis, MO, USA) and incubated at 37°C for 15 min of stimulation. Cells were washed twice with ice-cold phosphate-buffered saline (PBS) and lysed as described above. Equal amounts of protein (100 μg/lane) from each treatment were separated by SDS-PAGE (10% polyacrylamide gel) and transferred onto a PVDF membrane then blocked in blocking buffer at room temperature for 1 h. Primary antibodies to EGFR and p-EGFR were diluted 1:200 in blocking buffer at room temperature for 2 h and then with an appropriate secondary antibody at room temperature for 1 h. Parallel blots were probed under the same conditions with primary antibody β-actin diluted 1:1800 to confirm equal protein loading.

The relative intensities of band signals were determined by digital scanning densitometry and β-Actin was used to normalize the results to protein content.

### Statistical analysis

The data are presented as mean ± SD. Student's unpaired *t*-test was used for comparisons between treated and control cells and the level of statistical significance was set at *P *< 0.05 and *P *< 0.01.

## Results

### The effects of treatment with PUFAs on breast cancer cell growth

To evaluate the effects of PUFAs on breast cancer proliferation, cells were incubated for 3 days in medium supplemented with n-3 and n-6 PUFAs (EPA, DHA and AA).

**Figure 1:**
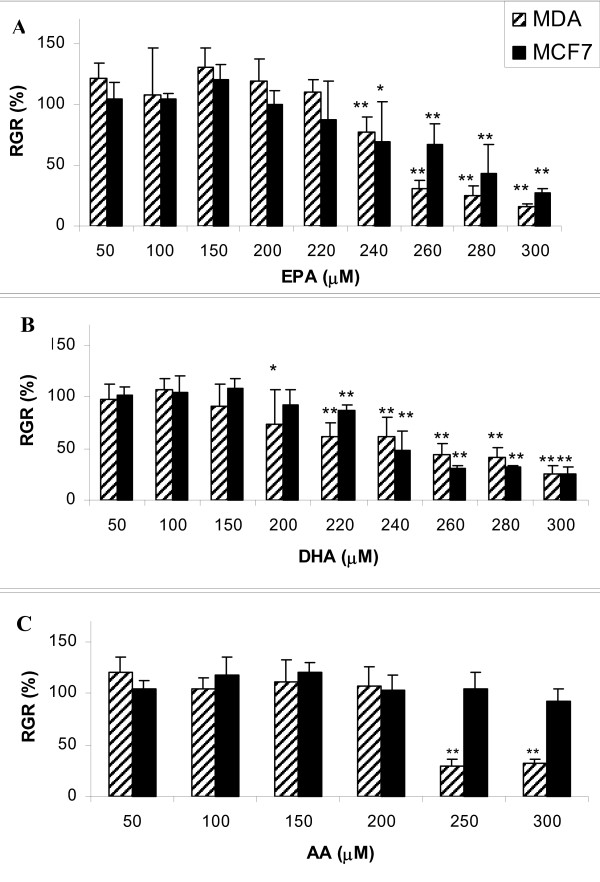
**Effects of PUFA on viability of breast cancer cells**. The effect on cell viability of PUFA in MDA-MB-231 and MCF7 cells is assessed and quantified by MTT assay. Cells are treated with various concentrations of EPA (A), DHA (B), and AA (C). Cells are seeded and cultured for 48 h in a 96-well plate, after this period, the medium is replaced with fresh medium for treatments with AA, EPA, or DHA and incubated for further 72 h. The numbers of viable cell exposed to fatty acids are evaluated by a colorimetric 3-(4,5-dimethylthiazol-2-yl)-2,5-diphenyltetrazolium bromide (MTT) assay. Data represent the mean of eight values and results are expressed as Relative Growth Rate (RGR) in comparison with controls (100%). * p < 0.05; ** p < 0.01 compared to control cells.

The effect on cell viability of MDA-MB-231 and MCF7 cells was assessed and quantified by the MTT assay. As shown in Figure 1, cells were treated with various concentrations of n-3 and n-6 PUFAs in the range 50-300 μM.

DHA and EPA induce a dose-dependent reduction of cell viability at concentrations > 200 μM (Figure 1A and 1B).

In contrast, AA (Figure 1C), the major n-6 PUFA, had no significant effect on MCF7 cell viability. The MCF7 cell line was more resistant than the MDA-MB-231 cell line to all treatments with PUFAs.

From these experiments, we extrapolated the dose to be used in successive experiments to assess n-3 PUFA incorporation into cell membrane PLs: 230 μM for EPA, 200 μM for DHA, which correspond to 70~80% viability for both cell lines, and 200 μM for AA.

### DHA and EPA induce apoptosis in breast cancer cells

**Figure 2:**
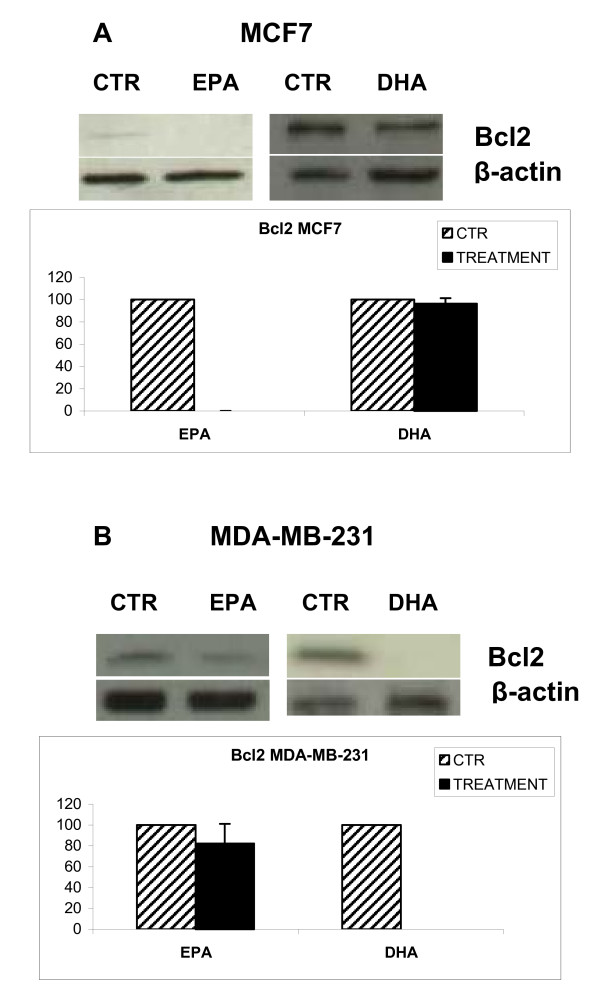
**Effects of n-3 PUFA on Bcl2 expression.** Both cell lines were treated with DHA (200 μM) or EPA (230 μM) for 72 h. Control and treated cell lysates are separated on 10% SDS-PAGE and transferred to PVDF membrane. The expression of the anti-apoptotic protein Bcl2 was assessed by western blot and  semi-quantitative analysis performed by plate scanning. β actin was used to normalize results of protein content. A: MCF7 cells, B MDA-MB-231 cells.

In order to delineate the possible mechanism(s) by which EPA and DHA induce apoptosis we examined the cytoplasmic levels of the Bcl2 protein. Figure 2A and 2B indicate that there was a slight reduction of Bcl2 level in MCF7 cells after treatment with 200 μM DHA, whereas treatment with 230 μM EPA determined the loss of signal; the expression of Bcl2 is also decreased when MDA-MB-231 cells are treated with 230 μM EPA and the protein is not detectable after incubation with 200 μM DHA.

Furthermore, apoptosis involves the activation of procaspase-8 (55 kDa) by its cleavage to caspase-8 (18 kDa); this smaller protein together with caspase-3 mediates the rapid dismantling of cellular organelles and architecture [28].

**Figure 3:**
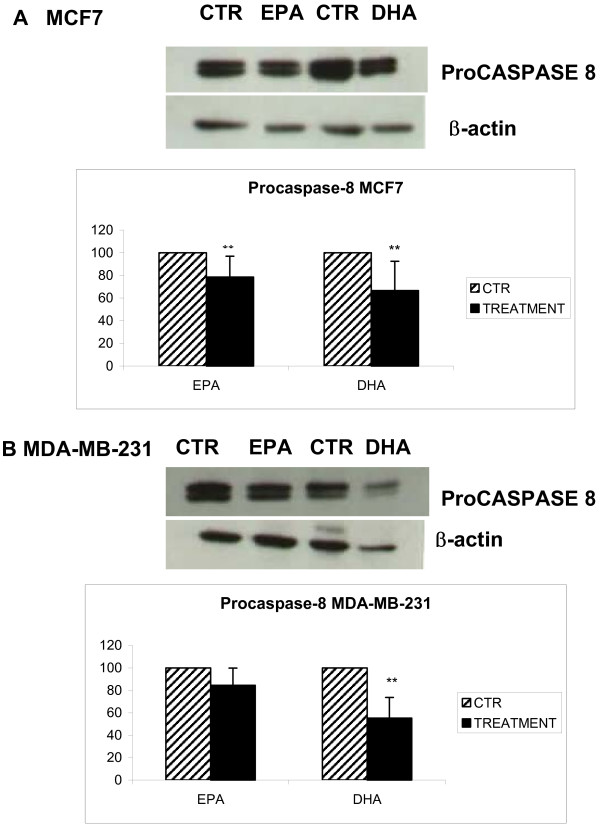
**Effects of n-3 PUFAs on caspase-8 expression**. The determination of the integrity of the procaspase-8 after treatment with DHA (200 μM) and EPA (230 μM) for 72 h was assessed in both cell lines. A: MCF7 cells treated with DHA (200 μM) or EPA (230 μM) for 72 h. B: MDA-MB-231 cells treated with DHA (200 μM) or EPA (230 μM) for 72 h. Semi-quantitative analysis performed by plate scanning. β-actin was used to normalize results of protein content. ** p < 0.01 compared to control cells; n = 3

The expression of procaspase-8 was determined by western blot analysis. In Figure 3A and 3B it is possible to observe a reduction of the proform of caspase-8 for both cell lines treated with EPA and DHA; the reduction was statistically significant after DHA treatment in both cell lines, and also after EPA treatment of MCF7 cells.

### EPA and DHA alter the EGFR and pEGFR levels in MDA-MB-231 cells

EGFR is usually activated in response to extracellular ligands (EGF) by its phosphorylation; ligand binding leads to homo- or heterodimerization with another ligand-bound ErbB receptor, and transmits extracellular mitogenic signals to downstream target signalling cascades that involve cell survival and proliferation, such as phosphatidylinositol 3-kinase (PI3K)/Akt, mitogen-activated protein kinase (MAPK) and signal transducer and activator of transcription 3 (STAT3) [29].

In human breast cancer cell lines, such as MDA-MB-231, the EGFR level is elevated compared with that in other breast cancer cells, such as MCF7 [30]; for this reason, we have studied the effects of DHA and EPA on EGFR activity mainly in MDA-MB-231 cells.

**Figure 4:**
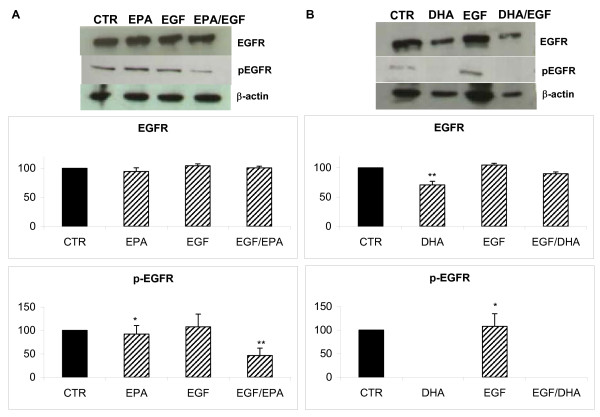
**EGFR expression and EGF stimulation (p-EGFR) in MDA-MB-231 breast cancer cells after n-3 PUFAs treatment**. A: MDA-MB-231 treated with 230 μM EPA for 72 h, lane 1 control, lane 2 230 μM EPA, lane 3 EGF, lane 4 EGF+EPA. B: MDA-MB-231 treated with 200 μM DHA for 72 h, lane 1 control, lane 2 200 μM DHA, lane 3 EGF, lane 4 EGF+DHA. The relative intensities of band signals, reported in graphics, are determined by digital scanning densitometry. β-actin was used to normalize results of protein content. * p < 0.05; ** p < 0.01 compared to control cells; n = 3.

Figure 4 reports the effects of EPA and DHA treatments on expression and activation of EGFR in presence of EGF. EPA did not modify EGFR expression in breast cancer cells; while EGF stimulation significantly increase EGFR phosphorylation to about 140%; co-treatment with EPA/EGF significantly inhibit EGFR activation down to about 40% compared to control non stimulated cells (Figure 4A).

As shown in Figure 4B, DHA significantly reduces the EGFR level (70%) compared to control cells and completely inhibit EGFR activation in cells treated with DHA or DHA/EGF.

### Total fatty acid profile after treatment with PUFA

Treatment with AA, EPA or DHA alters the FA profile in MDA-MB-231 and MCF7 cells compared with control cells (Table 1).

**Table 1:** Total fatty acid composition of PUFA-treated breast cancer cells

	MDA-MB-231				MCF-7			
	CTR	AA	EPA	DHA	CTR	AA	EPA	DHA
**C:16:0**	13.91 ± 2.19	13.48 ± 2.91	6.57 ± 0.91**	8.66 ± 1.25**	16.03 ± 4.06	15.52 ± 1.56	14.7 ± 1.76**	11.14 ± 3.59
**C 16:1**	1.61 ± 0.85	1.19 ± 0.87	0.87 ± 0.58**	0.77 ± 0.42*	8.25 ± 3.27	2.97 ± 0.86**	3.95 ± 0.54**	2.94 ± 1.53**
**C 18:0**	17.57 ± 1.97	10.46 ± 2.05*	5.15 ± 1.03**	6.39 ± 1.30**	14.83 ± 1.58	15.73 ± 1.76	10.41 ± 2.49**	9.46 ± 0.89**
**C 18:1**	26.14 ± 4.74	15.91 ± 4.21*	8.15 ± 0.95**	9.65 ± 1.29**	30.14 ± 2.91	12.12 ± 1.19**	12.21 ± 1.94**	9.77 ± 1.12**
**C 18:2**	6.05 ± 2.38	3.07 ± 0.65**	2.38 ± 0.17**	2.46 ± 0.32**	4.23 ± 0.73	2.48 ± 1.27**	2.26 ± 0.42**	1.66 ± 0.21**
**C 18:3**	1.54 ± 4.10	0.68 ± 0.37	0.22 ± 0.16	0.32 ± 0.10**	1.32 ± 0.87	0.96 ± 0.53*	0.59 ± 0.36*	0.63 ± 0.61*
**C 20:3**	1.64 ± 0.21	1.60 ± 0.10	0.72 ± 0.37**	1.18 ± 0.70*	2.41 ± 0.67	1.28 ± 0.11**	0.67 ± 0.09**	0.78 ± 0.16**
**C 20:4 (AA)**	14.40 ± 2.92	46.85 ± 10.48*	2.56 ± 0.87**	3.91 ± 0.50**	12.73 ± 2.90	44.26 ± 3.80**	3.13 ± 0.57**	3.20 ± 0.55**
**C 20:5 (EPA)**	2.42 ± 0.70	0.70 ± 0.23**	38.75 ± 3.52**	1.98 ± 0.43	3.22 ± 0.98	0.74 ± 0.75**	34.16 ± 3.89**	4.90 ± 0.51**
**C 22:5 (DPA)**	7.04 ± 1.10	3.61 ± 0.44**	33.30 ± 1.83**	2.94 ± 0.62**	1.10 ± 0.71	1.29 ± 0.45	15.80 ± 3.32**	0.96 ± 0.14
**C 22:6 (DHA)**	7.68 ± 1.55	2.45 ± 0.60**	1.29 ± 0.35**	61.76 ± 3.93**	5.73 ± 2.36	2.65 ± 0.68**	2.12 ± 0.31**	54.55 ± 6.02**
**SFA**	33.35 ± 5.50	23.94 ± 4.88*	11.76 ± 1.90**	15.05 ± 2.52**	30.86 ± 3.21	31.24 ± 2.89	25.11 ± 3.81**	20.60 ± 4.18**
**MUFA**	27.74 ± 4.69	17.10 ± 4.61*	9.01 ± 0.82**	10.41 ± 1.06**	38.39 ± 4.36	15.09 ± 1.62**	16.16 ± 2.02**	12.71 ± 2.27**
**n-3 PUFA**	18.68 ± 3.93	7.44 ± 0.56**	73.56 ± 3.16**	67.00 ± 3.45**	11.37 ± 3.71	5.63 ± 1.58**	52.67 ± 6.24**	61.05 ± 5.96**
**n-6 PUFA**	22.09 ± 3.15	51.52 ± 9.81**	5.67 ± 1.37**	7.55 ± 0.70**	19.38 ± 3.58	48.03 ± 4.00**	6.06 ± 0.76**	5.64 ± 0.59**

Breast cancer cells were treated with solvent (ethanol) as control, AA (20:4, n-6), EPA (20:5, n-3), and DHA(22:6, n-3).

Fatty acid composition was analyzed and expressed as percentage of total fatty acids (mean ± SD).

Asterisks indicate the significant differences between treated- and control cells (n=10, *P < 0.05; **P < 0.01).

Treatment of both cell lines with AA resulted in a significant increase of AA content in total cell lipids, from 14.40% to 46.85% in MDA-MB-231 and from 12.73% to 44.26% in MCF7. Furthermore, the data for MDA-MB-231 show a significant decrease of EPA, docosapentaenoic acid (DPA) and DHA, whereas the data for MCF7 cells show a significant reduction of only EPA and DHA.

When both cell lines were treated with EPA, the content of this FA in total cell lipids was increased significantly and we observed a significant reduction of AA. Unexpectedly, we found an increase of DPA content, indicating that EPA is incorporated into cells and is further metabolized by elongation.

The treatment with DHA determines a significant increase of DHA content in both cell lines and an increase of EPA content in MCF7, probably due to a retro conversion; a significant reduction of AA content was also measured.

### Effects of treatment with PUFAs on PL composition in breast cancer cells

Table 2 and 3 give the fatty acid composition of specific PLs in MDA-MB-231 and MCF7 cells treated with n-3 or n-6 PUFAs; to simplify the tables SD, are reported as plain numbers above the bold mean value.

**Table 2:** Phospholipids fatty acid composition of PUFA-treated MDA-MB-231

	C16:0	C16:1	C18:0	C18:1	C18:2	C18:3	C20:3	C20:4	C20:5	C22:5	C22:6	SFA	MUFA	PUFA	n-6 PUFA	n-3 PUFA	Omega-6/Omega-3	AA/EPA	AA/DHA
**PE CTR**	**5.13**	**0.80**	**19.58**	**18.11**	**3.12**	**0.62**	**1.58**	**30.38**	**3.65**	**6.14**	**7.85**	**24.71**	**18.90**	**53.34**	**35.08**	**18.26**	**1.95**	**8.96**	**3.93**
s.d.	1.87	0.59	2.40	2.59	0.97	0.19	0.37	4.39	0.90	1.01	1.06	3.16	2.80	4.03	3.42	2.01	0.30	2.99	0.79
**PE AA**	**6.16**	**1.29**	22.60*	15.64*	1.52*	0.30*	1.15*	40.72*	0.66*	3.75*	3.09*	28.76*	16.93*	51.08*	43.39*	7.70*	5.69*	81.49*	13.38*
s.d.	0.86	1.12	0.88	0.84	0.14	0.03	0.35	1.29	0.49	0.74	0.45	1.73	0.52	0.89	1.16	0.80	0.61	37.02	1.79
**PE EPA**	**5.64**	**0.55**	**20.44**	13.68*	1.66*	0.33*	0.84*	8.13*	22.52*	18.86*	1.62*	**26.09**	14.23*	**53.96**	10.63*	43.34*	0.25*	0.36*	**5.10**
s.d.	1.35	0.24	5.47	0.92	0.35	0.07	0.28	1.21	1.33	2.80	0.26	5.28	1.07	3.47	1.13	3.78	0.04	0.07	1.14
**PE DHA**	**6.44**	**1.14**	**19.87**	8.80*	1.34*	0.27*	0.84*	12.15*	2.10*	2.26*	42.31*	**26.31**	10.57*	**56.55**	13.46*	47.24*	0.26*	**6.41**	0.25*
s.d.	1.86	0.64	3.99	0.62	0.46	0.09	0.45	2.73	0.71	0.45	3.04	5.38	1.95	7.81	3.21	2.96	0.01	3.60	0.02

**PI CTR**	**4.32**	**1.31**	**34.32**	**14.48**	**2.50**	**0.50**	**2.88**	**25.54**	**1.44**	**4.65**	**3.98**	**40.05**	**14.35**	**41.67**	**30.34**	**11.33**	**3.23**	**22.84**	**8.98**
s.d.	1.73	1.31	5.38	4.35	1.74	0.35	0.66	3.93	0.65	1.12	1.74	5.37	5.32	5.09	6.33	3.34	1.68	11.79	9.39
**PI AA**	8.23*	**2.40**	**33.71**	**13.31**	1.44*	0.29*	0.65*	30.03*	0.40*	2.16*	**2.94**	**41.95**	**15.26**	**37.99**	**32.11**	5.87*	5.51*	75.95*	**10.23**
s.d.	2.67	1.24	2.05	1.54	0.40	0.08	0.30	2.86	0.04	0.15	0.36	3.09	2.39	2.53	2.70	0.40	0.69	15.180	1.75
**PI EPA**	6.16*	**1.12**	**24.38**	**14.81**	**2.34**	**0.47**	1.15*	11.53*	14.30*	16.40*	1.32*	30.55*	**15.93**	48.87*	15.02*	33.85*	0.45*	0.84*	**10.35**
s.d.	1.23	0.50	2.72	6.18	1.19	0.24	0.48	3.47	5.28	4.92	0.74	2.27	6.31	6.89	2.60	5.10	0.07	0.15	6.91
**PI DHA**	10.09*	**2.99**	**32.88**	**10.90**	**1.96**	**0.39**	1.81*	10.22*	0.96*	2.55*	20.43*	**42.96**	**13.89**	**39.71**	16.48*	22.57*	0.81*	10.77*	0.53*
s.d.	4.52	1.72	4.57	3.28	0.51	0.10	0.91	3.93	0.24	0.36	2.58	8.07	4.59	6.02	6.13	4.12	0.48	3.14	0.15

**PS CTR**	**6.22**	**2.16**	**26.07**	**23.76**	**3.69**	**1.10**	**2.27**	**13.12**	**2.50**	**5.74**	**7.52**	**32.40**	**24.94**	**38.00**	**20.12**	**16.73**	**1.25**	**6.03**	**1.90**
s.d.	1.88	1.96	9.75	6.53	1.62	1.07	0.99	7.67	1.20	1.69	3.71	9.19	7.87	11.64	10.49	4.72	0.68	3.59	1.64
**PS AA**	13.48*	4.69*	**21.00**	**25.00**	2.00*	0.48*	0.29*	**14.87**	0.41*	1.82*	**6.07**	**34.38**	**29.80**	26.37*	**17.81**	8.56*	2.12*	47.07*	**2.86**
s.d.	1.77	1.71	4.77	4.62	0.61	0.21	0.16	4.00	0.23	0.63	1.75	3.20	3.57	4.01	4.27	0.98	0.70	29.02	1.92
**PS EPA**	**6.17**	**1.38**	14.67*	**18.78**	1.76*	0.35*	**1.34**	6.96*	16.53*	23.17*	2.48*	**27.92**	**19.97**	**48.44**	10.06*	38.38*	0.29*	0.48*	**2.18**
s.d.	1.30	0.65	4.30	3.79	0.55	0.11	1.12	4.17	0.34	7.45	0.59	13.86	4.06	13.24	4.27	12.21	0.14	0.27	0.83
**PS DHA**	11.48*	**2.44**	**28.32**	10.43*	1.60*	0.32*	0.42*	**9.99**	1.23*	2.41*	30.84*	**36.77**	12.26*	**45.48**	**10.63**	34.49*	0.32*	**9.65**	0.33*
s.d.	3.46	1.48	13.14	1.56	0.29	0.06	0.13	2.22	0.40	0.83	8.78	17.15	3.15	11.23	3.27	8.40	0.09	3.81	0.05

**PC CTR**	**23.82**	**3.13**	**13.90**	**35.51**	**6.45**	**1.29**	**1.43**	**8.17**	**1.20**	**1.88**	**2.29**	**37.73**	**38.64**	**22.62**	**16.04**	**6.58**	**2.50**	**7.50**	**3.86**
s.d.	3.82	1.19	2.11	3.70	0.84	0.17	0.37	2.43	0.44	0.64	0.72	3.25	3.55	2.82	2.26	1.15	0.47	3.56	1.43
**PC AA**	27.29*	**3.84**	11.20*	21.03*	2.66*	0.53*	1.02*	27.61*	0.10*	1.45*	1.33*	**38.50**	24.87*	34.80*	31.29*	3.51*	8.89*	277.85*	18.21*
s.d.	1.51	0.42	0.69	1.98	0.18	0.04	0.13	3.19	0.01	0.23	0.28	1.43	2.03	3.27	3.13	0.15	0.56	34.12	2.17
**PC EPA**	**25.10**	1.63*	9.05*	20.73*	4.33*	0.87*	0.83*	3.62*	15.71*	14.26*	**1.36**	**34.14**	22.36*	40.96*	8.77*	32.17*	0.28*	0.24*	**3.42**
s.d.	3.84	0.25	1.94	1.79	0.66	0.13	0.42	0.53	2.09	5.43	0.93	4.74	1.92	6.92	0.36	6.75	0.05	0.04	1.29
**PC DHA**	**23.54**	**2.89**	**13.68**	16.85*	3.07*	0.61*	0.96*	**7.16**	1.78*	**1.80**	23.90*	**37.41**	20.77*	39.77*	11.80*	27.96*	0.43*	4.12*	0.33*
s.d.	2.72	0.88	1.65	3.35	0.37	0.08	0.25	0.98	0.45	0.66	4.15	4.53	1.62	3.56	0.42	3.75	0.08	0.54	0.09

**SM CTR**	**20.03**	**2.74**	**14.34**	**21.96**	**3.21**	**0.80**	**1.11**	**8.09**	**1.17**	**3.16**	**6.05**	**33.78**	**22.98**	**24.11**	**12.93**	**11.18**	**1.34**	**11.07**	**1.73**
s.d.	9.10	1.62	8.11	7.61	1.60	0.73	1.09	3.47	1.17	2.87	3.57	16.17	9.73	6.62	4.26	4.31	0.45	7.33	0.83
**SM AA**	**18.75**	**2.94**	**10.40**	**20.80**	**2.24**	**0.45**	0.53*	12.30*	**0.75**	0.63*	**6.68**	**33.32**	**23.22**	**22.58**	**14.80**	8.45*	1.76*	30.33*	**1.86**
s.d.	5.27	1.67	1.00	4.33	0.93	0.19	0.15	0.96	0.54	0.34	0.94	14.58	4.99	1.84	1.45	0.29	0.23	10.50	0.26
**SM EPA**	12.67*	**1.99**	6.43*	**24.50**	2.07*	**0.41**	0.38*	**7.85**	4.38*	6.28*	2.93*	19.11*	**24.70**	**26.51**	**13.17**	**16.24**	**0.90**	1.78*	**3.47**
s.d.	3.19	0.78	0.67	2.85	0.71	0.14	0.06	2.91	1.67	2.13	1.48	3.51	7.82	6.22	5.26	7.02	0.45	0.83	2.00
**SM DHA**	**19.07**	5.01*	**14.14**	13.62*	**2.89**	**0.58**	**1.72**	5.60*	**1.20**	1.24*	**8.35**	**33.21**	**19.47**	**25.69**	10.21*	**15.48**	0.79*	5.76*	0.70*
s.d.	6.89	0.79	3.32	1.01	0.42	0.08	0.73	1.88	0.53	0.23	2.52	9.19	2.32	7.32	1.37	7.31	0.37	3.21	0.37

Values are expressed as percentage of total fatty acids (means bold numbers, SD plain numbers).

CTR  n = 15, Treated n=6; * P < 0.01

**Figure 5:**
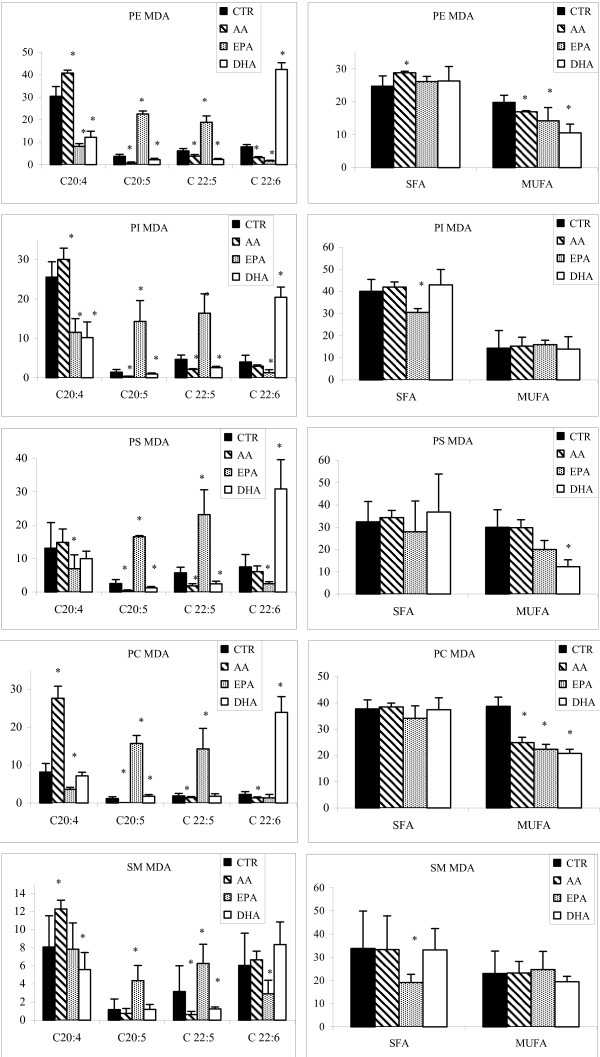
**Content of AA (C20:4), EPA (C20:5), DPA (20:6), DHA (C22:6), SFA and MUFA in PLs of MDA-MB-231 cells treated with LCPUFAs**. MDA-MB-231 cells were treated with AA, EPA, and DHA. Purification of single PL moieties was achieved with an HPLC-ELSD system. PL fatty acids were determined as methyl esters by gas chromatography (GC). Data are reported as percentage of total fatty acids. Controls are not exposed to any exogenous fatty acids. * p < 0.01; n = 3.

After treatment with AA, the content of this n-6 PUFA was increased significantly in PE and PC in MDA-MB-231 (Figure 5, Table 2). The treatment induced a reduction of omega-3 PUFAs, particularly EPA, that was significantly decreased in all phospholipids but SM; while DHA content was decreased after AA treatment only in PE and PC, the other two omega- 3 fatty acids namely DPA and ALA (C18:3) were decreased in all phospholipids but SM.

Incubation with EPA caused an increase of EPA content in all PLs in MDA-MB-231 cells. In particular, the incorporation of EPA was different in relation to the PL moiety with highest levels of incorporation in PI and PC. There was a decrease of monounsaturated FA in PE and PC, and a significant increase of polyunsaturated FA in PI and PC. Furthermore, an increase of DPA content was found in all PLs, especially PC. The content of AA was significantly decreased in all phospholipids but SM.

After treatment of MDA-MB-231 cells with DHA, the content of this fatty acid was significantly increased in all cell membrane PLs, but not in SM.

We measured a significant decrease of the content of EPA in PE, PS, PI and PC. The concentration of AA was significantly reduced in PE and PI and SM as the result of treatment with DHA.

**Figure 6:**
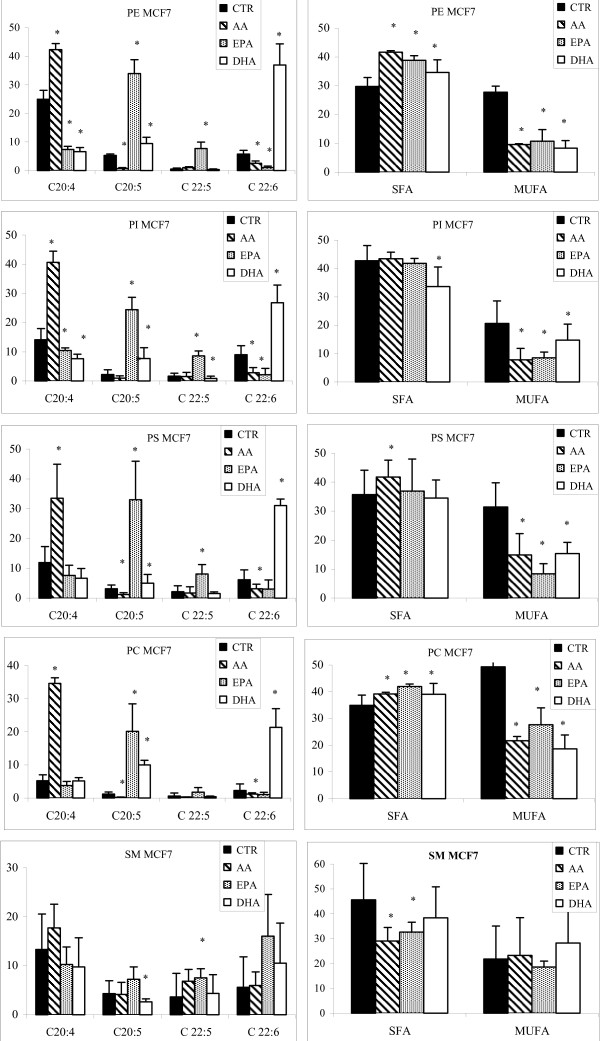
**Content of AA (C20:4), EPA (C20:5), DPA (20:6), DHA (C22:6), SFA and MUFA in PLs of MCF7 cells treated with LCPUFAs**. MCF7 cells were treated with AA, EPA, and DHA. Purification of single PL moieties was achieved with an HPLC-ELSD system. PL fatty acids were determined as methyl esters by gas chromatography (GC). Data are reported as percentage of total fatty acids. Controls are not exposed to any exogenous fatty acids. * p < 0.01; n = 3.

In MCF7 cells (Figure 6, Table 3), the treatment with AA induced a significant increase of this fatty acid in all PLs, except in SM; a significant reduction of EPA and of DHA in PE, PI, PS and PC was also measured.

**Table 3:** Phospholipids fatty acid composition of PUFA-treated MCF7

	C16:0	C16:1	C18:0	C18:1	C18:2	C18:3	C20:3	C20:4	C20:5	C22:5	C22:6	SFA	MUFA	PUFA	n-6 PUFA	n-3 PUFA	Omega-6/Omega-3	AA/EPA	AA/DHA
**PE CTR**	**7.76**	**4.23**	**22.01**	**23.50**	**3.46**	**0.69**	**1.70**	**24.93**	**5.28**	**0.72**	**5.71**	**29.77**	**27.73**	**42.50**	**30.09**	**12.41**	**2.47**	**4.75**	**4.58**
s.d.	1.18	1.91	1.92	3.14	0.69	0.14	0.28	3.13	0.69	0.56	1.37	1.86	3.83	3.88	2.66	1.96	0.37	0.50	1.04
**PE AA**	**8.24**	1.52*	33.44*	8.05*	1.38*	0.28*	0.49*	42.32*	0.70*	**1.03**	2.56*	41.68*	9.57*	48.75*	44.19*	4.57*	9.97*	76.64*	17.85*
s.d.	1.62	0.95	1.22	1.04	0.20	0.04	0.25	2.15	0.47	0.39	0.81	1.44	1.57	2.19	1.94	0.86	1.80	30.68	5.10
**PE EPA**	**9.52**	2.19*	27.35*	8.58*	1.50*	0.30*	0.53*	7.37*	33.91*	7.68*	1.07*	36.87*	10.77*	52.36*	9.40*	42.96*	0.23*	0.22*	**5.42**
s.d.	2.55	1.22	2.29	2.80	0.52	0.10	0.26	1.10	4.88	2.26	0.50	1.58	4.00	5.13	1.76	6.77	0.08	0.06	0.45
**PE DHA**	**9.26**	1.36*	**25.39**	6.97*	2.08*	0.42*	**1.03**	6.62*	9.45*	**0.52**	36.92*	34.65*	8.33*	57.02*	9.73*	47.30*	0.21*	0.71*	0.19*
s.d.	2.80	1.10	4.61	2.50	1.24	0.25	1.00	1.43	2.19	0.33	7.43	4.38	2.66	6.35	1.95	6.27	0.05	0.13	0.08

**PI CTR**	**10.84**	**2.51**	**31.95**	**18.13**	**3.43**	**0.69**	**5.50**	**14.10**	**2.22**	**1.64**	**8.99**	**42.79**	**20.65**	**36.56**	**23.02**	**13.54**	**1.80**	**8.04**	**1.84**
s.d.	5.40	2.32	5.42	6.55	1.29	0.26	1.53	3.89	1.65	1.00	3.07	5.35	7.91	7.50	4.66	3.77	0.47	4.32	1.20
**PI AA**	**8.22**	1.04*	35.22*	6.79*	1.45*	0.29*	1.01*	40.65*	0.96*	**1.52**	2.83*	**43.44**	7.83*	48.73*	43.12*	5.61*	8.34*	69.09*	23.36*
s.d.	1.78	0.73	2.36	3.69	0.34	0.07	0.13	3.84	0.80	1.41	1.77	2.35	4.03	2.81	3.70	1.65	2.63	49.53	17.92
**PI EPA**	**8.81**	**1.66**	**33.04**	6.85*	2.18*	0.44*	1.48*	10.41*	24.41*	8.60*	2.13*	**41.84**	8.51*	49.65*	14.07*	35.57*	0.40*	0.44*	**4.76**
s.d.	1.58	1.04	1.55	2.31	0.79	0.16	0.61	0.92	4.29	1.69	2.18	1.73	2.04	2.96	1.61	3.59	0.08	0.10	3.02
**PI DHA**	**9.06**	1.23*	24.60*	**13.54**	**4.52**	**0.91**	**3.11**	7.63*	7.71*	0.87*	26.82*	33.66*	14.77*	51.57*	15.26*	36.31*	0.43*	1.16*	0.31*
s.d.	3.81	0.91	5.96	5.74	2.54	0.51	3.09	1.55	3.67	0.76	6.04	6.91	5.63	7.46	5.64	5.29	0.19	0.48	0.17

**PS CTR**	**10.79**	**3.53**	**24.95**	**27.92**	**4.30**	**0.86**	**4.21**	**11.93**	**3.18**	**2.19**	**6.14**	**35.75**	**31.45**	**32.81**	**20.43**	**12.37**	**1.83**	**5.17**	**2.49**
s.d.	3.01	2.22	8.41	7.49	1.77	0.35	1.57	5.37	1.91	2.00	3.36	8.35	8.32	8.96	7.21	4.27	0.84	4.46	1.57
**PS AA**	**11.97**	**1.92**	**29.81**	12.97*	2.24*	0.45*	1.07*	33.50*	1.21*	**1.69**	3.18*	41.78*	14.89*	43.33*	36.80*	6.53*	6.49*	38.97*	12.22*
s.d.	4.28	2.83	7.69	4.92	1.33	0.27	0.43	11.48	0.63	2.12	1.47	5.84	7.35	9.32	10.79	2.15	3.45	31.62	6.48
**PS EPA**	16.78*	1.07*	**20.16**	7.31*	1.27*	0.25*	1.36*	**7.62**	33.04*	8.11*	**3.05**	**36.93**	8.37*	54.70*	10.25*	44.45*	0.22*	0.22*	**4.62**
s.d.	3.53	0.69	11.14	3.65	0.53	0.11	0.66	3.42	12.94	3.14	3.04	11.06	3.52	14.23	3.84	10.79	0.06	0.06	4.12
**PS DHA**	**9.83**	1.78*	**24.73**	13.57*	**3.40**	**0.68**	1.75*	6.67*	**5.00**	**1.55**	31.06*	**34.56**	15.35*	50.10*	11.82*	38.28*	0.32*	1.85*	0.22*
s.d.	3.89	1.47	6.84	3.83	1.70	0.34	1.30	3.26	2.94	0.59	2.17	6.22	3.88	4.33	5.70	4.53	0.18	1.50	0.12

**PC CTR**	**28.42**	**13.13**	**6.51**	**36.22**	**4.19**	**0.84**	**1.52**	**5.18**	**1.18**	**0.59**	**2.23**	**34.93**	**49.35**	**15.73**	**10.89**	**4.83**	**2.61**	**5.70**	**3.91**
s.d.	4.83	5.72	1.57	4.15	0.85	0.17	0.66	1.79	0.62	0.92	2.01	3.83	4.82	5.01	2.61	2.95	0.85	3.65	3.51
**PC AA**	**28.28**	4.97*	10.89*	16.67*	1.92*	0.38*	0.58*	34.61*	0.19*	**0.29**	1.21*	39.16*	21.65*	39.19*	37.11*	2.08*	18.12*	222.12*	30.38*
s.d.	2.59	1.87	2.23	0.69	0.05	0.01	0.05	1.68	0.10	0.07	0.33	0.63	1.61	1.91	1.61	0.33	2.44	100.56	8.64
**PC EPA**	**36.02**	**8.05**	**5.93**	19.61*	2.64*	0.53*	0.56*	**3.72**	20.12*	**1.75**	**1.07**	41.95*	27.66*	35.69*	6.92*	23.48*	0.34*	0.21*	**4.37**
s.d.	4.52	3.18	0.62	4.19	0.29	0.06	0.34	1.29	8.27	1.39	0.63	0.91	6.34	0.53	1.68	9.30	0.17	0.10	2.54
**PC DHA**	**29.09**	3.68*	9.94*	14.94*	3.37*	0.67*	**1.42**	**5.14**	10.01*	**0.39**	21.34*	39.03*	18.62*	42.35*	**9.93**	32.42*	0.32*	0.51*	0.26*
s.d.	5.46	2.60	1.71	3.86	0.46	0.09	0.39	0.99	1.36	0.20	5.63	4.11	5.19	6.44	1.47	6.01	0.07	0.06	0.08

**SM CTR**	**29.95**	**3.73**	**15.64**	**18.03**	**3.22**	**0.64**	**1.86**	**13.35**	**4.32**	**3.66**	**5.59**	**45.60**	**21.76**	**32.64**	**18.43**	**14.22**	**1.60**	**4.45**	**6.27**
s.d.	10.86	3.23	5.22	12.01	2.59	0.52	1.75	7.22	2.64	4.77	6.24	14.67	13.34	11.00	7.83	7.61	1.01	4.12	7.52
**SM AA**	17.58*	**2.89**	11.48*	**20.36**	**2.56**	**0.51**	**2.16**	**17.74**	**4.16**	**6.86**	**5.97**	29.06*	**23.25**	47.70*	**22.46**	**15.86**	**1.50**	**6.56**	**3.41**
s.d.	3.70	1.81	2.48	14.89	1.24	0.25	1.20	4.82	2.46	2.41	2.77	5.41	15.15	13.59	5.90	5.92	0.48	5.17	1.33
**SM EPA**	22.22*	13.52*	10.43*	5.06*	1.62*	0.32*	**5.71**	**10.28**	**7.24**	7.54*	**16.06**	32.65*	**18.57**	48.78*	**17.62**	31.16*	0.59*	1.49*	0.74*
s.d.	2.85	2.24	1.59	0.20	0.65	0.13	3.16	3.56	2.52	1.84	8.51	3.98	2.43	5.54	4.86	6.18	0.22	0.52	0.37
**SM DHA**	**27.05**	**5.19**	11.33*	**23.02**	**3.99**	**0.80**	**1.30**	**9.78**	2.65*	**4.38**	**10.53**	**38.38**	**28.21**	**33.41**	15.06*	**18.35**	**0.94**	**4.00**	1.05*
s.d.	10.74	4.69	2.93	20.63	1.93	0.39	0.89	5.93	0.60	3.81	8.19	12.47	18.30	15.33	6.22	11.75	0.41	2.60	0.47

Values are expressed as percentage of total fatty acids (means bold numbers, SD plain numbers).

CTR  n=5, Treated n=6; * P < 0.01

After treatment with EPA, the EPA and DPA content was significantly increased, especially in PE, PI, PS and PC. The concentration of DHA was significantly decreased in PE and PI, whereas the AA content was significantly reduced in PE, and PI.

The exposure of MCF7 cells to DHA determined a significant increase of DHA in all PLs, but not in SM, and of EPA content in PE, PI, and PC, whereas the content of AA was significantly reduced only in PE, PI and PS.

Also in these cells a significant decrease of monounsaturated fatty acids is always present when the cells are treated with n-6 and n-3 PUFAs; while saturated fatty acids are in most cases constant.

Moreover also 18:2, 18:3 (n-3) and 20:3 (n-6) are significantly decrease after PUFA treatment.

As far as phospholipids content concerns, it is worth noting the significant decrease of SM content (from 11.32% to 9.02%, data not shown) in MCF7 after treatment with DHA even if, sphingomyelin is the phospholipid less influenced in its fatty acid composition by PUFA treatment. The other treatments did not modify the distribution of PL in both cell lines.

## Discussion

Breast cancer is the leading cause of the death among women in the world. The principal effective endocrine therapy for treatment on this type of cancer is anti-estrogens, but therapeutic choices are limited for estrogen receptor (ER) negative tumor, which are more aggressive. Moreover the development of ER positive cancer cells that are resistant to chemotherapeutic agents is a major factor responsible to the successful treatment of breast cancer. This is a strong input to discover new approaches in vitro.

Several epidemiologic and clinical studies have shown that n-3 PUFAs are able to provide beneficial effects in a wide variety of pathologies ranging from autoimmune and inflammatory diseases to neurological and psychiatric disorders and, in particular, to several types of malignancy, including ovarian, pancreatic, prostate, renal, colorectal and breast cancer [31-33].

This study was prompted by the observation that MDA-MB-231 and MCF7 breast cancer cell lines showed a significant reduction in cell number following treatment with n-3 PUFAs. The same conclusion is not possible for the AA incubation. We hypothesize that this reduction in cell number results from both proliferation reduction and induction of apoptosis. Apoptosis is a genetically controlled form of cell death that is conserved from worms to humans. Deregulation of apoptosis is a hallmark of all cancer cells and the agents that activate apoptosis in cancer cells could be considered as anti-cancer therapeutics [34]. In some mammalian cells, apoptosis can be triggered by members of the Fas/TNF receptor family. When activated by receptor aggregation, Fas and TNFR1 induce the activation of a set of cysteine proteases called caspases. Studies designed to elucidate the mechanism(s) by which Fas and TNFR1 stimulation lead to caspase activation are underway. In the case of Fas, receptor aggregation by the Fas ligand induces the formation of a death-inducing signalling complex (DISC) of proteins comprising Fas itself, the adaptor protein FADD and the inactive zymogen form of caspase-8. Shortly after formation of the DISC, procaspase-8 is cleaved and the active protease is released. Once activated, caspase-8 is thought to activate other downstream caspases by proteolytic cleavage of their zymogen forms, thus amplifying the caspase signal [35]. Our results demonstrate the activation of caspase-8 in response to incubation with n-3 PUFAs by a reduction of the levels of its zymogen form in both cell lines. In many cells, over-expression of either Bcl2 or Bcl-xl inhibits apoptosis, affecting the release of cyt-*c *and apoptosis-inducing factor (AIF) from the mitochondrial intramembrane space to the cytosol. Once released, AIF is translocated to the nucleus where it is capable of inducing nuclear chromatin condensation and large-scale DNA fragmentation that mediates a caspase-independent mitochondrial apoptotic pathway [36]. Cyt-*c*, together with dATP, binds to apoptotic proteinase activating factor-1 (Apaf-1) and this complex promotes procaspase-9 autoactivation. The active forms of caspase-8 and caspase-9 might activate the downstream effectors caspase-3, -6 and -7, resulting in the cleavage of crucial cellular proteins and apoptosis. We have observed a significant difference in the amount of Bcl2 present in the DHA-treated MDA-MB-231 cells and EPA-treated MCF7 cells compared to the control group. The absence of Bcl2 when compared to the control is suggestive that the cell might be more likely to proceed to apoptosis.

EGFR is an interesting target for tumour therapy, because it is over-expressed in many human tumours such as lung and breast cancers [37]. MDA-MB-231 cells express high levels of EGFR and are a good model to study EGFR modulation by n-3 PUFAs. This receptor is a member of the ErbB receptor tyrosine kinase family, which consists of EGFR (or HER1 or ErbB1), HER2/ErbB2, HER3/ErbB3 and HER4/ErbB. Ligand binding to EGFR induces its dimerization with another EGFR or with other members of the ErbB family, and activates tyrosine kinase residues on the intracellular domains of the protein through autophosphorylation. EGFR recruits downstream signalling proteins, triggering signal cascades along a number of pathways that eventually lead to cell growth, migration and apoptosis resistance [38,39]. We found that the phosphorylated EGFR levels are reduced after treatment with n-3 PUFAs (EPA or DHA) in MDA-MB-231 cells, whereas the EGFR level was decreased only after incubation with DHA.

The entire mechanism by which n-3 PUFAs exert their beneficial effects is not fully understood. We have hypothesized that the induction of apoptosis, the reduction of cell proliferation and the inhibition of EGFR activity by these fatty acids might be the consequences of cell membrane alterations induced by FA. Our data indicate that EPA and DHA are incorporated in breast cancer membrane. In particular the EPA treatment determines an increase of EPA and DPA content, and a reduction of SFA, MUFA and n-6 PUFA concentration in both cell lines. This suggests an incorporation of EPA which is further metabolised. In fact, EPA is converted to 22:5 n-3 (DPA) by elongase (Elovl)-5 and then by Elovl-2 to 24:5, n-3. The next step requires desaturation of 24:5 by Δ6 desaturase to produce 24:6, n-3. This product is translocated from the endoplasmic reticulum to the peroxisome, where the β oxidation pathway involves acyl chain shortening of C2 to produce DHA [40].

Also DHA incubation determines an increase of EPA, of DHA, and in general of the unsaturation degree in both cell lines. We have also observed that PUFAs are incorporated into the breast cancer membrane with different specificity for each PL moiety. The enrichment is significant, especially in PE, PI and PC. The transbilayer distribution of lipids across biological membranes is asymmetric. The choline-containing lipids PC and SM are enriched primarily on the external leaflet of the plasma membrane or the topologically equivalent luminal leaflet of internal organelles. In contrast, the amine-containing glycerophospholipids PE and PS are located preferentially on the cytoplasmic leaflet. Other minor PLs, such as phosphatidic acid (PA), PI and phosphatidylinositol-4,5-bisphosphate (PIP_2_) are also enriched on the cytofacial side of the membrane. Specific alterations of the molecular composition of the plasma membrane occur during apoptosis. Hence, cells undergoing apoptosis express signals, including lipids, proteins and modified sugar moieties that facilitate recognition and ingestion by macrophages. Loss of transmembrane PL asymmetry, with consequent exposure of PS in the external monolayer, occurs in both normal and pathologic conditions. PS externalization is induced early in the process of apoptosis. On the basis of our findings, the data suggest that the incorporation of n-3 PUFAs is mainly into cytofacial leaflet PLs, altering the membrane environment to impact on the activation of cell signalling. Moreover, a significant decrease of SM was evident in cells treated with DHA.

Once lipid asymmetry has been established, it is maintained by a combination of slow transbilayer diffusion, protein-lipid interactions and protein-mediated transport. The most significant contributors to the maintenance and dissipation of transbilayer lipid asymmetry are proteins that catalyse the movement of lipids across the membrane. Two classes of transport activities have been described that are responsible for the ATP-dependent transport of lipids. The best characterized activity is flippase, which transports PS from the outer monolayer to the cytoplasmic surface of the plasma membrane and requires ATP and Mg^2+ ^but its activity is inhibited by Ca^2+^. A second ATP-dependent activity, catalysed by flippases, transports lipids in the opposite direction. The third class of lipid transporter consists of the Ca^2+^-activated scramblases that catalyse the PS externalization [41,42]. Growing evidence indicates that excessive concentrations of FA affect cell functions by altering the activity of various ion transporters and channels, including Ca^2+^. Zhang *et al. *have found that PUFAs, but not monounsaturated or saturated FAs, cause [Ca^2+^]_i _mobilization in NT2 human tetracarcinoma cells by causing release of this proton from mitochondria [43]. Furthermore, Djemli-Shipkolye *et al. *showed that FA modifications in membranes could be correlated with the variations observed in the activity of ATPase, for instance of Mg-ATPase [44]. This effect could influence the flippase and scramblase activities, and thus the transbilayer lipid asymmetry.

Moreover PUFA incorporation induces an alteration of SFA, MUFA and PUFA content in membrane phospholipids; these data suggest a metabolic rearrangement in cells in order to try to balance the ratio between saturated and unsaturated fatty acids.

In addition membranes constitute a meeting point for lipids and proteins. Thousands of cellular proteins interact with membranes in different ways, for example integral (transmembrane, as EGFR) proteins are embedded in the lipid bilayer and their activity is sensitive to changes in the lipid environment. Recently, multiple studies demonstrated very rapid ERα actions at level of the plasma membrane [45]. O'Malley and collaborators have demonstrated that ERα on the membrane initially activates cytoplasmic kinases, which in turn phosphorylate and activate coactivators proteins in the cytoplasm. These coactivators then travel to the nucleus and modulate ERα-mediated transcriptional events [46].

Then n-3 PUFAs, modifying the unsaturated degree, the permeability, the flip-flop process and the fluidity of the plasma membrane, might alter the activity of these proteins. This hypothesis will be investigated in our laboratory.

## Conclusions

We suggest that n-3 PUFAs induce modifications of membrane structure and function of breast cancer cells, thereby increasing the degree of unsaturation. These changes of plasma membrane might modify the membrane architecture and signal transduction causing a reduction of cell proliferation and apoptosis induction.

## Abbreviations

PL: (phospholipid); FA: (fatty acid); PUFA: (polyunsaturated fatty acid); MUFA: (monounsaturated fatty acid); SFA: (saturated fatty acid); DHA: (docosahexaenoic acid); EPA: (eicosapentaenoic acid); AA: (Arachidonic acid); PE: (phosphatidylethanolamine); PI: (phosphatidylinositol); PC: (phosphatidylcholine); PS: (phosphatidylserine); SM: (sphingomyelin).

## Competing interests

The authors declare that they have no competing interests.

## Authors' contributions

PAC carried out cell treatments, MTT tests, WB assays and drafted the manuscript, GM performed lipid analysis, SZ was responsible for cell cultures, IEJ performed lipid analysis, AC performed lipid analysis, BB Coordinated the study, AMR conceived and designed the study, performed analysis and interpretation of data and drafted the manuscript.

All authors have read and approved the final manuscript.
